# A comparison of the video head impulse test and the functional head impulse test in chronic unilateral vestibular loss

**DOI:** 10.1017/S0022215123001536

**Published:** 2024-01

**Authors:** Gulce Kirazli, Hatice Seyra Erbek

**Affiliations:** 1 Department of Audiology, Faculty of Health Sciences, Ege University, Izmir, Turkey; 2 ENT Clinic, Lokman Hekim University/Akay Hospital, Ankara, Turkey

**Keywords:** Vestibular function tests, head impulse test, dizziness, vertigo, vestibular diseases

## Abstract

**Objective:**

To examine the correlation of video head impulse test, functional head impulse test and Dizziness Handicap Inventory results in patients with chronic unilateral vestibular loss, and to compare the results with healthy controls.

**Methods:**

Forty-eight patients diagnosed with chronic unilateral vestibular loss and 35 healthy individuals, aged 18–65 years, were included. The video head impulse test, functional head impulse test and Dizziness Handicap Inventory were administered.

**Results:**

A significant positive correlation was found between functional head impulse test and video head impulse test results for the study group in all semicircular canals (*p* < 0.05). There was no significant correlation between Dizziness Handicap Inventory, functional head impulse test and video head impulse test results (*p* > 0.05). The functional head impulse test and video head impulse test results of the control group were significantly higher than those of the study group in all semicircular canals planes (*p* < 0.05).

**Conclusion:**

In chronic unilateral vestibular loss patients, with high head accelerations, the functional head impulse test indicates deterioration in vestibulo-ocular reflex functionality. It would be beneficial to include the video head impulse test and functional head impulse test in clinical practice as complementary tests in vestibulo-ocular reflex evaluation.

## Introduction

In patients with chronic unilateral vestibular loss, vestibular diagnosis becomes difficult as the central nervous system activates effective adaptive mechanisms for recovery. Especially in patients with chronic unilateral vestibular loss, eye movements elicited by the rotational vestibulo-ocular reflex often succeed in stabilising the visual field during angular acceleration of the head, taking advantage of the modulation of the firing rate of the afferents provided by healthy semicircular canals.^1^ An example is the caloric test, which evaluates the vestibulo-ocular reflex in both ears for low frequency (0.003–0.05 Hz) stimuli.^2^

In daily life, when we move our head under normal conditions, the vestibular labyrinths are stimulated in the frequency range 0.1–5 Hz.^2^ In real life, therefore, high-frequency vestibulo-ocular reflex has a more important role in visual stabilisation, especially during daily activities.^3^ The video head impulse test can show the distortions more clearly, as it evaluates the vestibulo-ocular reflex at high frequencies (>1 Hz) and high angular head accelerations (1000–4000°/s^2^). Even if a patient has normal caloric responses, vestibulo-ocular reflex distortions and low vestibulo-ocular reflex gain can be seen during head movements in a high-frequency range in the video head impulse test.^4–6^

As an objective measure that quantitatively measures the vestibulo-ocular reflex, the video head impulse test does not provide direct information on the functional effectiveness of the motor response, the latter of which provides good gaze stabilisation and thus clear vision. In other words, the video head impulse test provides a match rate that indicates whether the vestibulo-ocular reflex elicits the desired eye movement, but it does not give the clinician an idea of whether the patient uses eye movements for clear vision during head movement, and thus meet their functional purpose in vestibulo-ocular reflex responses.^7^

In the functional head impulse test, which was developed in line with the functional purpose of the vestibulo-ocular reflex response, there is no need to calculate the reflex gain; in addition, it is easier to use, less costly and can be utilised in a large number of patients without eye movements being recorded. This test is based on the patient's ability to recognise the optotype displayed on the computer screen during head rotations and calculation of the percentage of correct answers.^3,8^

Although there is an increase in vestibulo-ocular reflex gain in chronic unilateral vestibular loss, the role of eye movements in correct reading ability and residual vestibulo-ocular reflex functionality during rapid head rotations in the horizontal and vertical semicircular canal planes is unclear.

The present study aimed to: evaluate different angular head accelerations in all semicircular canals with the functional head impulse test and the video head impulse test in patients with chronic unilateral vestibular loss; reveal the relationship between the functional head impulse test percentage of correct answers and video head impulse test gain; compare the results with those of healthy controls; and examine the correlation of the Dizziness Handicap Inventory results with the functional head impulse test and video head impulse test results.

## Materials and methods

### Participants

This cross-sectional and prospective study was carried out in the Baskent University Neuro-otology Clinic of the Department of Otorhinolaryngology and Head and Neck Surgery, Ankara/Turkiye between July 2019 and March 2020.

The study was approved by the Medical and Health Sciences Research Board (project number: KA19/177) and the Non-Interventional Clinical Research Ethics Committee (approval date, 22 May 2019; approval number, 19/67). A signed informed consent form for scientific research was obtained from all individuals participating in the study.

The study sample size was determined by power analysis. According to the calculation made using the G*power 3.1 program, the total sample size was determined to be at least 68, with an effect size of 0.80, margin of error of 0.05, confidence level of 0.95 and population representation of 0.90. A total of 83 participants, 48 in the study group (patients with chronic unilateral vestibular loss) and 35 in the control group (healthy participants), were included.

In order to make the chronic unilateral vestibular loss diagnosis, after the patient's history was taken by an ENT specialist and following an examination, vestibular function tests (videonystagmography and bithermal caloric test), audiological tests (pure tone audiometry, speech audiometry and tympanometry) and magnetic resonance imaging were performed. In 18–65-year-old patients with vestibular symptoms persisting for more than 3 months, and in whom unilateral canal paresis of at least 25 per cent was obtained as a result of the bithermal caloric test, a diagnosis of chronic unilateral vestibular loss was made by the ENT specialist. Patients were excluded from the study if they had: positive oculomotor findings (from saccadic, pursuit, optokinetic and positional tests), bilateral vestibular disease (vestibulopathy), a central vestibular disorder, semicircular canal dehiscence, benign positional paroxysmal vertigo, post-traumatic vertigo and chronic otitis, conductive hearing loss and middle-ear pathology, severe visual impairment, severe neurological and cognitive problems (under 24 points in the Standardized Mini-Mental State Examination), or could not be tested for various reasons (neck problems, eye problems).

Thirty-five participants with normal hearing and no balance or other health problems, and who presented to the clinic for routine ENT examination, were included in the control group.

The video head impulse test, the functional head impulse test and the Dizziness Handicap Inventory were administered to the study group, and only the video head impulse test and functional head impulse test were administered in the control group. The tests were completed on the same day.

### Video head impulse test

The video head impulse test was administered with an EyeSeeCam video head impulse test device (A/S DK-5610; Interacoustics, Assens, Denmark). Special goggles (type-1085 ICS Impulse®) were used, with a monocular camera mounted on the left or right side and a laser source placed in the middle. All participants (patients and healthy volunteers) put on the goggles; they were then seated upright, 1.5 m from the target point on the wall, and their eye level was adjusted. Once eye and head calibrations had been completed, the test phase was started.

The test phase included the evaluation of lateral and vertical semicircular canals. The researcher stood behind the participant and grasped the participant's chin with both hands. At least 10 impulses were made to the right and left randomly and quickly (faster than 150°/s), at 15–20° angles, by tilting the participant's head forward by 30°. The head position was placed back to the midline after a few seconds. In the evaluation of the vertical canals, the right anterior and left posterior semicircular canals were tested together, and the left anterior and right posterior semicircular canals were tested together as they were located in the same plane. For the left anterior and right posterior semicircular canals, the participant's head was turned approximately 35–45° to the right while their body still faced straight ahead, and for the right anterior and left posterior semicircular canals, the participant's head was turned approximately 35–45° to the left. The researcher grasped the patient's chin with one hand and placed the other hand on the patient's head. The participants were asked to look steadily at the target on the wall ahead. Standing behind the participant, the researcher moved the patient's head up and down unexpectedly and rapidly (faster than 150°/s) at an angle of about 10–20°. At least 10 impulse moves were made for each canal.

The vestibulo-ocular reflex gain values of all participants were recorded with the velocity regression gain measurement method, in the plane of the right and left lateral and vertical (anterior and posterior) semicircular canals.

### Functional head impulse test

The functional head impulse test was carried out using a Beon Solutions (Zero Branco, Italy) functional head impulse test system, consisting of a computer with a software program, a gyroscope (headband sensor) and a mini keyboard. The gyroscope (headband sensor) was used to measure the angular velocity and direction of the head, and was placed in the middle of the forehead.

Before starting the test, participants' static visual acuity was assessed. During this phase, while the participants sat in front of the computer monitor without moving their heads, the Landolt C optotype flashed very quickly on the monitor in one of eight different directions at a time.

At the beginning of the static visual acuity test, the size of the Landolt C optotype was 1.0 LogMAR (log of the minimum angle of resolution), but after three correct responses to the Landolt C optotype in each of five different directions, the optotype size was reduced. Thus, based on participants’ correct and incorrect answers, the minimum readable optotype suitable for each participant was obtained in LogMAR through the ‘quest algorithm’. After static visual acuity was determined in this way and the minimum threshold value for each participant had been found, LogMAR was increased by 0.6 and brought to the level that the person could see most comfortably. The dynamic testing phase was then started.

The size of the Landolt C optotype, which was six lines greater than the best static visual acuity, remained constant throughout the dynamic testing phase. Head stimuli during the dynamic test consisted of fast (faster than 150°/s), passive, random direction and low amplitude (±20°) head movements in both directions (a minimum of 10 movements). Before each head movement, the participant was asked to look at the target white dot appearing in front of a black background on the monitor and wait. The participant was asked to recognise and distinguish the direction of the vanishing optotype among the Landolt C optotypes in eight different directions using the mini keyboard. There was no time limit for the test.

In the testing phase of the lateral canals, while the participant was sitting at a distance of 1.5 m directly in front of the monitor, the researcher stood behind the participant, tilted their head 30° forward and grasped their chin with both hands. Random left and right head impulse movements were performed and the head was returned to the midline after a few seconds of waiting.

While testing the left anterior and right posterior canals, the participants' body was rotated 45° to the left, and during the right anterior and left posterior testing the participants' body was rotated 45° to the left. Participants were asked to look directly at the monitor and turn their head towards the monitor so that they could clearly see the optotypes on the screen. Thus, the visual axis and the head axis were aligned. The tester performed up and down head impulses at the sagittal plane of the participants' body, standing across from the participant and grasping one hand above the head and the other under the chin.

**Figure 1.**
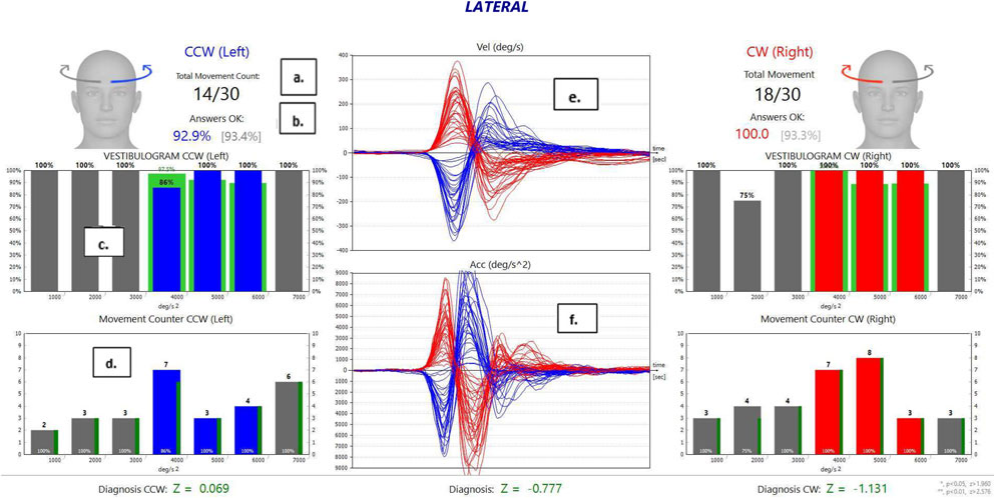
Functional head impulse test results of the right and left lateral semicircular canals of a healthy participant. ‘(a.)’ Indicates the total number of head impulses performed in the left and right directions out of 30 movements. ‘(b.)’ Shows the total percentage of correct answers in the head acceleration range 4000–6000°/s^2^. ‘(c.)’ Refers to the percentage of correct answers obtained in each acceleration (1000–7000°/s^2^). ‘(d.)’ Highlights the number of head impulses applied in each acceleration. ‘(e.)’ Shows a head velocity graph (red, right ear; blue, left ear). ‘(f.)’ Shows a head acceleration graph (red, right ear; blue, left ear). CCW = counterclockwise; CW = clockwise

Because the functional head impulse test evaluates the functionality of the vestibulo-ocular reflex in different head accelerations, the head impulses applied are classified according to their direction (right and left) in acceleration steps (step, 1000°/s^2^; range, 2000–7000°/s^2^), and online feedback is provided (Figure 1). A graphical user interface reports the number of impulses performed per acceleration step and the corresponding percentage of correct answers, thus helping the clinician to decide the head impulse acceleration rate.^1,9^

**Figure 2.**
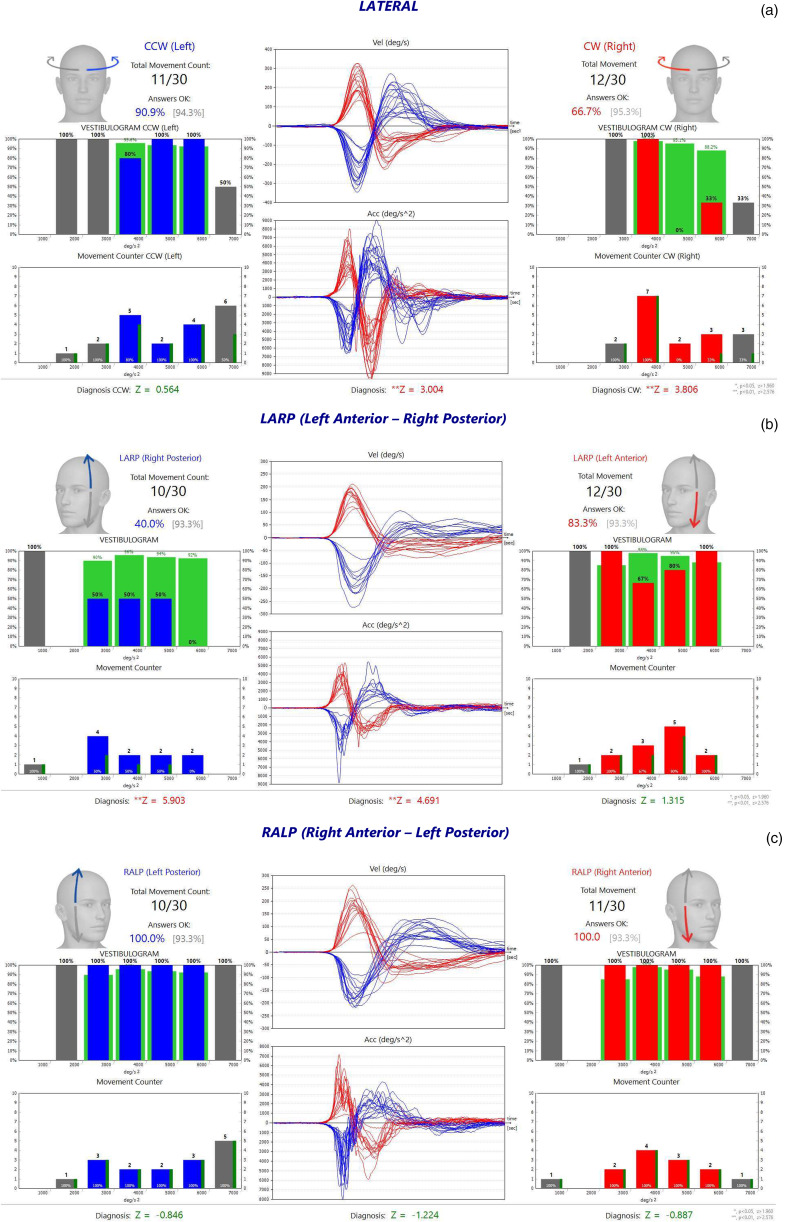
Functional head impulse test results in lateral and vertical semicircular canals for a patient with chronic right-sided vestibular loss and diagnosed with Ménière's disease: (a) lateral, (b) left anterior, right posterior, and (c) right anterior, left posterior semicircular canal types. CCW = counterclockwise; CW = clockwise

The mean percentage of correct answers in lateral semicircular canals in the head acceleration range of 4000–6000°/s^2^, the mean percentage of correct answers in the head acceleration range of 3000–6000°/s^2^ in vertical (anterior and posterior) semicircular canals, and the mean percentage of correct answers for head acceleration steps of 3000, 4000, 5000 and 6000°/s^2^ separately for all semicircular canals, were recorded and analysed (Figure 2). Thus, the correlation between head acceleration rate and the percentage of correct answers was also examined. The percentage of correct answers obtained in head accelerations outside the head acceleration range of 3000–6000°/s^2^ was not included in the analysis. The total percentage of correct answers obtained in the test was considered to reflect high-frequency dynamic visual acuity.

### Dizziness Handicap Inventory

The Dizziness Handicap Inventory subjectively measures the disability level associated with dizziness and balance problems. A validity and reliability study was conducted in Turkish by Karapolat *et al*.^10^ The Dizziness Handicap Inventory evaluates the subjective disability level with 25 items and a total of 100 points. For each question in the scale, patients are asked to mark one answer: ‘yes’, ‘no’ or ‘sometimes’.

### Statistical analysis

Data analysis was carried out with SPSS® version 25 statistical software. The significance level (*p*) for the comparison tests was set to 0.05. Tests were performed to check the conformity of the data with the normal distribution and to determine which of the parametric and non-parametric methods would be used. Whether or not the data included in the study conformed to the normal distribution was checked separately for each group using the Kolmogorov–Smirnov test.

Given that the assumption of normality was ensured in the analyses performed, the analyses were continued with parametric test methods. The difference between the genders of the two groups was analysed with the chi-square test. The significance test (*t*-test) of the difference between the two means was used for comparisons between paired groups. The homogeneity of variance was checked with Levene's test when deciding which test result to use in comparisons (*p* > 0.05). Correlation analysis was performed to assess the relationship between measurement values. The Pearson correlation co-efficient was used because the data were distributed normally.

## Results

The study comprised 48 participants with chronic unilateral vestibular loss and 35 healthy participants. In the study group, the affected side (48 ears) was analysed. Both ears of 35 participants in the control group were included in the statistical analysis (70 ears).

The mean age of the participants in the study group was 48.42 years. Of the 48 ears included in the study group, 56.3 per cent were from females and 43.8 per cent were from males. Overall, 48.5 per cent of participants had canal paresis in the right ear and 54.2 per cent had canal paresis in the left ear. The average rate of canal paresis was 64.18 per cent. The mean onset of disease symptoms was 40.04 months.

The mean age of the control group was 46.06 years; 65.7 per cent of the participants were female and 34.3 per cent of the participants were male. There was no statistically significant difference between the two groups in terms of age or gender (*t* = 0.826, *p* = 0.411; *χ*^2^ = 0.757, *p* = 0.384).

The mean Dizziness Handicap Inventory total score of the study group was 45.42.

In the lateral, anterior and posterior semicircular canal planes, the study group had a significantly lower total percentage of correct answers and lower mean percentage of correct answers at the 3000, 4000, 5000 and 6000°/s^2^ steps compared to the control group (*p* < 0.05) for the functional head impulse test (Tables 1–3).

**Table 1.** Percentage of correct answers in lateral semicircular canal study group versus control group

Variable	Group	Mean ± SD	Levene*	Test value	*p*-value
Total CA%	Study group	65.88 ± 31.04	0.001	−7.368	0.001^†^
	Control group	94.27 ± 7.38			
CA% for head accelerations of 3000°/s^2^	Study group	82.35 ± 29.62	0.001	−4.128	0.001^†^
	Control group	97.87 ± 8.89			
CA% for head accelerations of 4000°/s^2^	Study group	72.85 ± 32.53	0.001	−5.912	0.001^†^
	Control group	96.94 ± 8.6			
CA% for head accelerations of 5000°/s^2^	Study group	66.19 ± 35.66	0.001	−6.528	0.001^†^
	Control group	95.56 ± 10.12			
CA% for head accelerations of 6000°/s^2^	Study group	54.56 ± 38.67	0.001	−5.462	0.001^†^
	Control group	88.43 ± 22.59			

*Levene test; variances are homogeneous. ^†^*p* < 0.05, *t*-test for difference of the means between the two samples. SD = standard deviation; CA% = percentage of correct answers

**Table 2.** Percentage of correct answers in anterior semicircular canal study group versus control group

Variable	Group	Mean ± SD	Levene*	Test value	*p*-value
Total CA%	Study group	74.8 ± 23.9	0.001	−5.869	0.001^†^
	Control group	92.91 ± 8.14			
CA% for head accelerations of 3000°/s^2^	Study group	80.94 ± 28.17	0.001	−4.855	0.001^†^
	Control group	98.11 ± 7.66			
CA% for head accelerations of 4000°/s^2^	Study group	78.58 ± 27.31	0.001	−5.200	0.001^†^
	Control group	96.74 ± 8.71			
CA% for head accelerations of 5000°/s^2^	Study group	70.77 ± 33.97	0.001	−3.867	0.001^†^
	Control group	89.19 ± 17.3			
CA% for head accelerations of 6000°/s^2^	Study group	58.21 ± 37.76	0.001	−4.364	0.001^†^
	Control group	83.63 ± 25.56			

*Levene test: variances are homogeneous. ^†^*p* < 0.05, *t*-test for difference of the means between two samples. SD = standard deviation; CA% = percentage of correct answers

**Table 3.** Percentage of correct answers in posterior semicircular canal study group versus control group

Variable	Group	Mean ± SD	Levene*	Test value	*p*-value
Total CA%	Study group	72.57 ± 24.67	0.001	−5.619	0.001^†^
	Control group	91.12 ± 10.35			
CA% for head accelerations of 3000°/s^2^	Study group	80.58 ± 25.10	0.001	−4.316	0.007^†^
	Control group	95.34 ± 11.43			
CA% for head accelerations of 4000°/s^2^	Study group	76.23 ± 31.66	0.001	−4.439	0.001^†^
	Control group	94.49 ± 11.26			
CA% for head accelerations of 5000°/s^2^	Study group	69.58 ± 31.99	0.001	−4.783	0.001^†^
	Control group	90.79 ± 15.62			
CA% for head accelerations of 6000°/s^2^	Study group	52.08 ± 38.12	0.013	−4.168	0.015^†^
	Control group	78.71 ± 31.06			

*Levene test: variances are homogeneous. ^†^*p* < 0.05, *t*-test for difference of the means between two samples. SD = standard deviation; CA% = percentage of correct answers

The mean vestibulo-ocular reflex gain values in all semicircular canal planes of the affected side of the study group were significantly lower than those of the control group (*p* < 0.05) for the video head impulse test (Table 4).

**Table 4.** Vestibulo-ocular reflex gain value in all semicircular canals between the study group and the control group

Variables	Group	Mean ± SD	Levene*	Test value	*p*-value
Lateral semicircular canal	Study group	0.84 ± 0.24	0.001	−5.066	0.001†
	Control group	0.99 ± 0.07			
Anterior semicircular canal	Study group	1.06 ± 0.26	0.002	−2.908	0.004†
	Control group	1.17 ± 0.17			
Posterior semicircular canal	Study group	1.02 ± 0.31	0.001	−4.406	0.001†
	Control group	1.22 ± 0.18			

*Levene test: homogeneity of variances. ^†^*p* < 0.05, *t*-test for difference of the means between two samples. VOR = vestibulo-ocular reflex; SD = standard deviation

In the lateral and posterior semicircular canals, a moderate significant positive correlation (*r* = 0.678 and *r* = 0.487, respectively) was found between the mean functional head impulse test total percentage of correct answers of the study group and the mean video head impulse test vestibulo-ocular reflex gain value (*p* = 0.001). In the anterior semicircular canal, a statistically significant weak positive correlation was found between the mean functional head impulse test total percentage of correct answers and the mean video head impulse test vestibulo-ocular reflex gain value (*r* = 0.341, *p* = 0.018; Table 5).

**Table 5.** Relationship between percentage of correct answers and gain values in all semicircular canal types

Correlations	semicircular canal plane	*R* value*	*p*-value
Correlation between CA% of study group & gain	Lateral	0.678	0.001^†^
	Anterior	0.341	0.018^†^
	Posterior	0.487	0.001^†^
Correlation between CA% of control group & gain	Lateral	0.138	0.255
	Anterior	0.159	0.188
	Posterior	0.061	0.617

*Pearson correlation co-efficient. ^†^*p* < 0.05. CA% = percentage of correct answers

For the control group, no statistically significant correlation was found between the mean functional head impulse test total percentage of correct answers and the mean video head impulse test vestibulo-ocular reflex gain value, in all semicircular canals (lateral *p* = 0.255, anterior *p* = 0.188 and posterior *p* = 0.617, respectively; Table 5).

There was no statistically significant correlation between the total percentage of correct answers, the vestibulo-ocular reflex gain and the Dizziness Handicap Inventory total score of the study group in the lateral, anterior and posterior semicircular canals (*p* > 0.05).

## Discussion

In the literature, there are limited studies, especially involving the functional head impulse test, on the functional consequences of the vestibulo-ocular reflex in chronic unilateral vestibular loss.^1,3,8,11^ Unlike the studies in the literature, the present study evaluated the functional deterioration of the vestibulo-ocular reflex in vertical semicircular canals as well as lateral semicircular canals. In addition, in all semicircular canals, the quantitative integrity of the vestibulo-ocular reflex pathway, and the relationship of subjective vertigo with the perception of disability, were examined in chronic unilateral vestibular loss patients, and the test results were compared with a control group.

### Vestibulo-ocular reflex functionality in rapid head acceleration

This section examines the evaluation of vestibulo-ocular reflex functionality in rapid head acceleration in chronic unilateral vestibular loss. In our study, as the head acceleration increased in all semicircular canals, the percentage of correct answers started to decrease in the study group. Especially in the lateral semicircular canal, a significant difference was found between the mean percentage of correct answers in head accelerations in the 3000 and 4000°/s^2^ steps of the affected ears. In higher head accelerations (5000 and 6000°/s^2^), the mean percentage of correct answers was lower on the affected side. This result shows that, in patients with chronic unilateral vestibular loss, as head acceleration increases, vestibular function is non-linear and deterioration in vestibulo-ocular reflex functionality is more clearly seen. Thus, we believe that the functional head impulse test can provide insight into the amount of impairment.

The total percentage of correct answers in the control group was found to be over 90 per cent in all semicircular canals. As head acceleration increased, the percentage of correct answers decreased. On the other hand, there was a statistically significant difference between the total percentage of correct answers in the control group and the total percentage of correct answers in the study group in each semicircular canal. In addition, the percentage of correct answers in the control group and the study group differed significantly in all head accelerations.

Emekci and Erbek evaluated the correlation between the functional head impulse test and age in healthy adults.^12^ Similar to our study, in all semicircular canals, they found that the total percentage of correct answers was close to 90 per cent, and the percentage of correct answers decreased as head acceleration increased.

In the study by Romano *et al*., which determined the functional head impulse test normative values specific to different types of sport in the horizontal and vertical semicircular canal planes, the results for professional athletes and healthy non-athletes were compared.^9^ There was no significant difference between the two groups in terms of the total percentage of correct answers, which was above 90 per cent for both groups. Accordingly, this study reported that a typical healthy individual is expected to give nearly 100 per cent of correct answers in the functional head impulse test. Thus, it was suggested that the functional head impulse test may increase the specificity of vestibular disorders given that the percentage of correct answers ratio is correctly determined by the functional head impulse test in healthy individuals.

Apart from the present study, no study evaluating the vertical canal plane with regard to chronic unilateral vestibular loss using the functional head impulse test was found in the literature. However, a limited number of studies evaluating vertical dynamic visual acuity in this group are available.

In a study conducted with the dynamic visual acuity test, the vertical plane result of a group diagnosed with unilateral vestibular hypofunction was not found to be significantly different from that of healthy normal participants.^13^ In our study, there was a significant difference between the percentage of correct answers and the total percentage of correct answers, in all head accelerations (from low to high), between the control group and the study group, in posterior and anterior semicircular canals.

In a study conducted by Roberts and Gans, in which they compared horizontal and vertical dynamic visual acuity test findings in patients with unilateral vestibular dysfunction, results similar to our study were obtained.^14^ Although the horizontal dynamic visual acuity score is twice as sensitive as the vertical dynamic visual acuity score in demonstrating the deterioration of vestibulo-ocular reflex functionality, 14 out of 33 patients showed a decrease in the dynamic vertical plane. In the control group, normal dynamic visual acuity scores were found in both planes. As unilateral vestibular dysfunction was diagnosed according to the results of the caloric test in the study, the importance of testing the vertical canals was also emphasised.

When the inferior vestibular nerve is damaged, the caloric test result may be normal, while the posterior video head impulse test result is abnormal. When there is damage to the main body of the vestibular nerve, the video head impulse test gain in all canals may be low.^15^ It is therefore suggested that evaluating only the horizontal semicircular canal in patients may miss the functional impairment of the vestibulo-ocular reflex in the vertical plane, and adversely affect the diagnosis and the subsequent treatment process. It was noted that a therapy designed to focus solely on gaze stabilisation in the horizontal plane may not allow adequate gaze stabilisation in the vertical plane, and this may not become apparent unless the vertical vestibulo-ocular reflex task is performed.^14^

The importance of evaluating function in both planes after treatment to demonstrate the appropriate outcome has been suggested.^14^ In line with these results, we think that the evaluation of the vertical semicircular canal in patients diagnosed with chronic unilateral vestibular loss will play an important role in determining vestibulo-ocular reflex functionality that may be overlooked in this plane, which may result in insufficiency in developing an appropriate therapy plan.

### Correlation of functional and video head impulse test results

Although the vestibulo-ocular reflex gains of the study group in the video head impulse test were within normal limits in all canals, the mean total percentage of correct answers in the functional head impulse test was below the normal values reported in the literature. This result is in line with the study of Corallo *et al*,^3^ who obtained the mean vestibulo-ocular reflex gain of the affected side in the horizontal semicircular canal plane of patients with vestibular neuronitis close to the normal range (0.70) three months after the disease. However, these authors detected more abnormalities in the functional head impulse test on the affected and healthy sides, both in the acute phase and three months later, than in the video head impulse test.

Corallo *et al*. also did not find any correlation between the video head impulse test and the functional head impulse test findings.^3^ It was emphasised that although these results indicate that the compensation mechanism increases the gain values, this finding is not sufficient to read the target correctly during passive head movement.

Ramat *et al*. emphasised that while patients with covert saccades may have more normal results in the video head impulse test, lower scores can be obtained during the functional head impulse test because functionally the patient cannot see clearly.^8^ Different assumptions regarding the discordance between the two results have been discussed. Accordingly, the first assumption is that the vestibulo-ocular reflex gain does not exactly match the vestibulo-ocular reflex adequacy. The video head impulse test provides an objective assessment of vestibulo-ocular reflex behaviour according to a mathematical model, but it does not provide direct information about the functional efficiency of the motor response. Second, there are many external factors that affect the outcome of the functional head impulse test; for example, results are affected not only by the ability of the angular vestibulo-ocular reflex to produce an effective protective motion to identify the ‘C’ optotype, but also by the ability to recognise it. This ability, in turn, depends on the functional integrity of both optical pathways and cognitive mechanisms. Thus, the fact that the functional head impulse test is not conditioned by vestibular mechanisms alone may explain the inconsistency of the results with those of the video head impulse test.^16^

In our study, unlike other studies, a significant positive correlation was found between the functional head impulse test results and the video head impulse test results in all semicircular canal planes on the affected side of the study group. Although vestibulo-ocular reflex deterioration is more clearly seen in the functional head impulse test than the video head impulse test, an increase or decrease in the percentage of correct answers, especially on the side of vestibular weakness, indicates that there will be an increase or decrease in video head impulse test gains.

The video head impulse test and functional head impulse test findings show that normal vestibulo-ocular reflex gain is a necessary, but not sufficient, condition to guarantee effective gaze stabilisation during angular accelerations of the head at high frequencies. In the light of these findings, the video head impulse test and functional head impulse test should be considered as two complementary tests for vestibulo-ocular reflex assessment. While the video head impulse test assesses the integrity of the vestibulo-ocular reflex pathway by measuring the vestibulo-ocular reflex response, the functional head impulse test provides information on vestibulo-ocular reflex functionality, which can be affected even by non-vestibular factors.^16^

### Perception of dizziness handicap, and vestibulo-ocular reflex functionality and integrity

This section examines the relationship between the subjective perception of dizziness handicap, vestibulo-ocular reflex functionality, and vestibulo-ocular reflex integrity. There are studies in the literature that have examined the correlation between caloric test results, video head impulse test results and Dizziness Handicap Inventory results in unilateral peripheral vestibular disorders. Redondo-Martínez *et al*. evaluated the relationship between results of the caloric test, video head impulse test and Dizziness Handicap Inventory in patients with vestibular neuritis in the acute phase and three months after the onset of symptoms, and could not find a significant linear correlation between the test results.^17^ In a study by McCaslin *et al*., in patients with a history of vestibular neuritis or labyrinthitis, similarly, no correlation was found between perceived dizziness handicap, caloric asymmetry and video head impulse test results.^18^ Corallo *et al*.^3^ and Versino *et al*.^19^ found no significant correlation between horizontal canal video head impulse test and functional head impulse test parameters and Dizziness Handicap Inventory scores in patients with vestibular neuritis.

- The video head impulse test does not provide direct information on the functional effectiveness of the motor response, which provides good gaze stabilisation and thus clear vision
- The functional head impulse test was developed in line with the functional purpose of the vestibulo-ocular reflex response
- According to our results, the functional head impulse test indicates deterioration in vestibulo-ocular reflex functionality in chronic unilateral vestibular loss patients
- The results suggest that the functional head impulse test and the video head impulse test should be included in clinical practice
- These could act as complementary tests in vestibulo-ocular reflex assessment for chronic unilateral vestibular loss

In our study, similar to the literature, no significant correlation was obtained between the functional head impulse test total percentage of correct answers and video head impulse test gain values, and the Dizziness Handicap Inventory total score in all semicircular canals. While the healing process of canal paresis takes a long time, and inconsistency with the Dizziness Handicap Inventory is expected, considering that the video head impulse test and the functional head impulse test contain movement frequencies similar to rapid head movements in daily life, the lack of a significant relationship between these tests and the Dizziness Handicap Inventory is intriguing.

Given the inconsistency of the objective and subjective test results, Redondo-Martínez *et al*. stated that these tests do not appear to be indicative of the patient's subjective clinical status. Therefore, a clear relationship could not be established between the caloric test, the video head impulse test, the functional head impulse test and the Dizziness Handicap Inventory, emphasising that the Dizziness Handicap Inventory is a subjective test evaluating parameters that are difficult to standardise, for example dizziness and imbalance disorders.^17^ McCaslin *et al*. also stated that unless vestibular disorders are bilateral and severe, objective and subjective test results are generally inconsistent with each other.^20^

The consensus emerging in the literature is that subjective assessment scores of dizziness-related disabilities are more strongly associated with factors other than objective test abnormalities. The inconsistency between the objective vestibular test results and Dizziness Handicap Inventory scores has been explained as due to: the Dizziness Handicap Inventory scores being affected by different variables, for example patients’ anxiety and depression levels; differences in coping strategies; the duration of dizziness and illness; and cognitive and emotional factors.^20–22^ In addition, some factors – for example lifestyle, the general health status of the person, family support level, the motivation to cope with physical illnesses, and stress – cannot be controlled.^21^ For this reason, it is emphasised that the level of perceived handicap cannot be explained only by the presence of the underlying structural vestibular disorder, and may result from a combination of impaired visual-vestibular perception and psychological characteristics.^23^ We therefore believe that the use of other psychometric tools assessing anxiety, depression and psychological stress, as well as scales assessing subjective disability perception, for example the Dizziness Handicap Inventory, will make a comprehensive contribution to the measurement of patients’ subjective symptoms.

## Conclusion

The results of our study show that the functional head impulse test and the video head impulse test should be included in clinical practice as complementary tests in the vestibulo-ocular reflex assessment. In addition, as factors such as anxiety, depression and stress, which increase the subjective perception of handicap, may affect objective test results, additional use of psychometric measurement tools may provide a more comprehensive assessment. In future studies, in addition to the evaluation of vestibulo-ocular reflex functionality with the functional head impulse test in patients with chronic unilateral vestibular loss, it will be necessary to examine the relationship between the video head impulse test and the presence of covert and overt saccades (e.g. saccade latency and amplitude) and vestibulo-ocular reflex gains, to determine the vestibular compensation mechanism process.
